# Rho-Kinase 1/2 Inhibition Prevents Transforming Growth Factor-β-Induced Effects on Pulmonary Remodeling and Repair

**DOI:** 10.3389/fphar.2020.609509

**Published:** 2021-01-20

**Authors:** Xinhui Wu, Vicky Verschut, Manon E. Woest, John-Poul Ng-Blichfeldt, Ana Matias, Gino Villetti, Alessandro Accetta, Fabrizio Facchinetti, Reinoud Gosens, Loes E. M. Kistemaker

**Affiliations:** ^1^ Department of Molecular Pharmacology, Faculty of Science and Engineering, University of Groningen, Groningen, Netherlands; ^2^ Groningen Research Institute for Asthma and COPD, University Medical Center Groningen, University of Groningen, Groningen, Netherlands; ^3^ AQUILO BV, Groningen, Netherlands; ^4^ Corporate Pre-Clinical R and D, Chiesi Farmaceutici S.p.A., Parma, Italy

**Keywords:** pulmonary remodeling, lung repair, rock inhibition, lung organoid, TGFβ signaling

## Abstract

Transforming growth factor (TGF)-β-induced myofibroblast transformation and alterations in mesenchymal-epithelial interactions contribute to chronic lung diseases such as chronic obstructive pulmonary disease (COPD), asthma and pulmonary fibrosis. Rho-associated coiled-coil-forming protein kinase (ROCK) consists as two isoforms, ROCK1 and ROCK2, and both are playing critical roles in many cellular responses to injury. In this study, we aimed to elucidate the differential role of ROCK isoforms on TGF-β signaling in lung fibrosis and repair. For this purpose, we tested the effect of a non-selective ROCK 1 and 2 inhibitor (compound 31) and a selective ROCK2 inhibitor (compound A11) in inhibiting TGF-β-induced remodeling in lung fibroblasts and slices; and dysfunctional epithelial-progenitor interactions in lung organoids. Here, we demonstrated that the inhibition of ROCK1/2 with compound 31 represses TGF-β-driven actin remodeling as well as extracellular matrix deposition in lung fibroblasts and PCLS, whereas selective ROCK2 inhibition with compound A11 did not. Furthermore, the TGF-β induced inhibition of organoid formation was functionally restored in a concentration-dependent manner by both dual ROCK 1 and 2 inhibition and selective ROCK2 inhibition. We conclude that dual pharmacological inhibition of ROCK 1 and 2 counteracts TGF-β induced effects on remodeling and alveolar epithelial progenitor function, suggesting this to be a promising therapeutic approach for respiratory diseases associated with fibrosis and defective lung repair.

## Introduction

Fibroblast to myofibroblast differentiation represents an essential event during wound closure and tissue repair. Transforming growth factor (TGF)-β plays a major role in promoting myofibroblast differentiation. However, excessive and persistent TGF-β-induced myofibroblast differentiation and extracellular matrix (ECM) deposition contribute to pathological tissue remodeling that occurs in a broad range of lung diseases, such as chronic obstructive pulmonary disease (COPD) (Grzela et al., 2016), asthma (Fehrenbach et al., 2017), and idiopathic pulmonary fibrosis (IPF) (King et al., 2011; Hirota and Martin, 2013; Ohgiya et al., 2017). Myofibroblasts are contractile cells possessing morphologic and biochemical features that are intermediate between fibroblast and smooth muscle cells. These contractile fibroblasts secrete ECM proteins such as collagens, which are the most important load-bearing component of the parenchymal lung connective tissue, crucial for maintaining structural and mechanical organ functionality (El Agha et al., 2017). Moreover, the differentiated myofibroblasts are characterized by enhanced expression of α-smooth muscle actin (α-SMA) and other cytoskeletal proteins contributing to the contractile activity of these cells (Meng et al., 2016; Florian et al., 2019; Winters et al., 2019).

As such, the persistent presence of myofibroblasts in disease may actually contribute to defective repair by airway and alveolar epithelial cells. Mesenchymal-epithelial interactions normally contribute to epithelial regeneration after injury, yet myofibroblasts are less effective in supporting epithelial repair (Demayo et al., 2002; Horowitz and Thannickal, 2006; Meng et al., 2016). Previously, we reported that TGF-β-induced myofibroblast differentiation profoundly skews the canonical WNT/β-catenin signaling in human lung fibroblasts, and results in reduced secretion of factors that nurture epithelial repair such as FGF7, FGF10 and HGF (Ng-Blichfeldt et al., 2019). Furthermore, TGF-β increases the expression of WNT-5A and WNT-5B by myofibroblasts (Ng-Blichfeldt et al., 2019), and such mesenchymal WNT-5A/5B signaling represses alveolar epithelial repair by inhibition of canonical WNT signaling (Wu et al., 2019a). Currently, there are no pharmacological treatments available in the clinic that effectively prevent or reverse the aberrant TGF-β-induced changes in remodeling and lung repair.

Rho-associated coiled-coil containing kinases (ROCK) are part of the AGC (cAMP-dependent protein kinase/protein kinase G/protein kinase C) kinase family that play crucial roles in several vital cellular functions including gene transcription, proliferation, differentiation, and apoptosis (Klein et al., 2019; Majolée et al., 2019; Park et al., 2019; Wang et al., 2019). Two isoforms, ROCK1 and ROCK2, were described as being part of this family of RhoA-GTP interacting proteins. Several studies have revealed a diverse range of functions of ROCK1 and 2 in the context of lung diseases (Htwe et al., 2017) and ROCK inhibitors have potential therapeutic applicability in lung diseases such as asthma, COPD and pulmonary fibrosis (Hallgren et al., 2012; Vigil et al., 2012; Htwe et al., 2017; dos Santos et al., 2018; Knipe et al., 2018). A recent study also showed that the gene expression of both ROCK1 and ROCK2 were increased in the lungs of the patients who died from Covid-19 (Ackermann et al., 2020). Pharmacological inhibition of ROCK using ROCK inhibitors has been shown to prevent airway remodeling and lung fibrosis in animal models (Qi et al., 2015; Knipe et al., 2018). Fasudil, a classic non-selective ROCK inhibitor and vasodilator approved in Japan for the treatment of brain vessel vasospasm induced by subarachnoid haemorrhage, is reported playing protective roles in bleomycin-induced pulmonary fibrosis in animal models (Jiang et al., 2012); however, its clinical applications are limited by the modest ROCK inhibition efficacy and poor selectivity (Tumbarello and Turner, 2006; Hallgren et al., 2012; Pireddu et al., 2012; Rath and Olson, 2012; Vigil et al., 2012; Xueyang et al., 2016; Huang et al., 2018).

The differential role of ROCK isoforms on TGFβ signaling in fibrosis and repair has not been thoroughly investigated, yet. To fill this gap, we have selected from existing patents two potent ROCK-inhibitors: compound 31, a dual ROCK1 and ROCK2 inhibitor, and compound A11, a ROCK2 selective inhibitor. We then evaluated their efficacies in three *in vitro* models to identify their potential in restoring TGF-β-induced changes in myofibroblast differentiation and impaired alveolar epithelial progenitor cell function. Our results show that dual ROCK1 and 2 inhibition prevents myofibroblast differentiation and ECM deposition induced by TGF-β in lung fibroblasts and PCLS, whereas ROCK2 selective inhibition did not. Furthermore, our results reveal that dual ROCK1/2 and ROCK2 inhibition restores the defective TGF-β-induced changes in mesenchymal-epithelial progenitor interactions during organoid formation, suggesting ROCK inhibition as a promising therapeutic target for pulmonary diseases characterized by defective lung repair.

## Materials and Methods

### Synthesis of compound 31 and compound A11

3,4-dimethoxy-N-((R)-1- (3 - (((S) - 6 - (propylamino) - 4, 5, 6, 7 tetrahydrobenzo [d]thiazol-2-yl) carbamoyl) phenyl) ethyl) benzamide (example 31 from WO 2012/006202) and N-(3-(5-((4-chloro-1H-indazol-5-yl)amino)-1,3,4-thiadiazol-2-yl)phenyl)-1-methyl-1H-pyrazole-4-carboxamide (example A11 from WO 2016/138335) were prepared by adapting the general synthesis procedures reported in the patent references.

### Inhibition of ROCK1 and ROCK2 Enzymatic Activity

ROCK enzymatic activity inhibition was measured as described previously (Cantoni et al., 2019). Glutathione S-transferase (GST)-tagged 1–535 human ROCK1 and GST-tagged 1–552 human ROCK2 (Fisher Scientific United Kingdom Ltd., Loughborough, Leicestershire, United Kingdom) were diluted into assay buffer containing 40 mM Tris pH7.5, 20 mM MgCl2 0.1 mg/ml BSA, 50 μM DTT and 2.5 μM peptide substrate (myelin basic protein). Compounds to be tested were dissolved in dimethyl sulphoxide (DMSO) to a final concentration of 1%. All reactions/incubations were performed at 25°C. The compounds and either ROCK1 or 2 were mixed and incubated for 30 min. Reactions were initiated by addition of ATP (10 μM). After a 1 h incubation, 10 μl of ADP-Glo Reagent (Promega United Kingdom Ltd., Southampton, United Kingdom) was added and after another 45 min incubation, 20 μl of kinase Detection Buffer were added and then the mixture was incubated for 30 min. The luminescent signal was measured on a luminometer. Compounds were tested in a dose-response format. To determine the IC50, data were fit to a plot of % inhibition vs. Log10 compound concentration with a sigmoidal fit using activitybase software (v 8.05, ID Business Solutions Limited, Guildford, United Kingdom).

### Animals

Animals were housed conventionally under a 12-h light-dark cycle and received food and water ad libitum. All experiments were performed in accordance with the national guidelines and approved by the University of Groningen Committee for Animal Experimentation (license numbers: AVD10500201581 and AVD105002015303).

### Precision-Cut Lung Slices

PCLS were harvested from 8–12 week old female Balb/c mice as described previously (Oenema et al., 2013; Van Dijk et al., 2017; Wu et al., 2019b). Briefly, animals were euthanized by subcutaneous injection of ketamine (40 mg/kg, Alfasan, Woerden, The Netherlands) and dexdomitor (0.5 mg/kg, Orion Pharma, Mechelen, Belgium), and the trachea was exposed and cannulated. Lungs were filled with 1.5 ml of 1.5% low melting-point agarose solution (Gerbu Biotechnik GmbH, Wieblingen, Germany) in CaCl_2_ (0.9 mM), MgSO_4_ (0.4 mM), KCl (2.7 mM), NaCl (58.2 mM), NaH_2_PO_4_ (0.6 mM), glucose (8.4 mM), NaHCO_3_ (13 mM), Hepes (12.6 mM), sodium pyruvate (0.5 mM), glutamine (1 mM), MEM-amino acids mixture (1:50), and MEM-vitamins mixture (1:100), pH = 7.2). Agarose was allowed to solidify at 4°C for 15 min and lungs were then harvested. Lobes were separated and sliced individually, at a thickness of 250 µm in medium composed of CaCl_2_ (1.8 mM), MgSO_4_ (0.8 mM), KCl (5.4 mM), NaCl (116.4 mM), NaH_2_PO_4_ (1.2 mM), glucose (16.7 mM), NaHCO_3_ (26.1 mM) andHepes (25.2 mM), set at pH = 7.2, using a tissue slicer (Leica VT1000S, Vibratome line, Amsterdam, The Netherlands). Slices were transferred in cell culture dishes, at 37°C in a humidified atmosphere of 5% CO_2_ and medium (CaCl_2_ (1.8 mM), MgSO_4_ (0.8 mM), KCl (5.4 mM), NaCl (116.4 mM), NaH_2_PO_4_ (1.2 mM), glucose (16.7 mM), NaHCO_3_ (26.1 mM), Hepes (25.2 mM), sodium pyruvate (1 mM), glutamine (2 mM), MEM-amino acids mixture (1:50), MEM-vitamins mixture (1:100) penicillin (100 U/mL) and streptomycin (100 μg/ml), pH = 7.2.) was refreshed every 30 min for four times to remove any remaining agarose and cell debris.

### Treatments on PCLS

PCLS were incubated in Dulbecco’s Modification of Eagle’s Medium (DMEM) supplemented with sodium pyruvate (1 mM), MEM non-essential amino acid mixture (1:100; Gibco® by Life Technologies), gentamycin (45 μg/ml; Gibco® by Life Technologies), penicillin (100 U/mL), streptomycin (100 μg/ml), and amphotericin B (1.5 μg/ml; Gibco® by Life Technologies) at 37°C-5% CO_2_ in a 12-well plate (three slices per well). Slices were treated with vehicle, 2 ng/ml TGF-β_1_ (2 ng/ml, R&D systems, Abingdon, United Kingdom) and/or investigational compounds (Table 1) for 48 h. The PCLS were then stored at −80°C until PCR analysis, Western Blot analysis.

**TABLE 1:** Investigational compounds and concentrations in the current study.

Compound	IC50 ROCK1	IC50 ROCK2	Concentration	Selectivity
Compound 31	2.9 ± 0.5 nM	4 ± 0.6 nM	0.01, 0.1 and 1 μM	ROCK1 and 2
Compound A11	341.1 ± 38 nM	6.1 ± 1.4 nM	0.1, 1 and 10 μM	ROCK2 selective

### Fibroblast Cell Culture and Treatments

Human lung fibroblasts MRC5 (CCL-171; ATCC, Wesel, Germany) were cultured in Ham’s F12 medium (Life technologies, Carlsbad, United States) supplemented with 10% (v/v) fetal bovine serum (FBS, PAA Laboratories, Pasching, Austria), 2 mM l-glutamine (Life Technologies #35050–061), 100 U/mL penicillin/streptomycin, and 1% amphotericin B (1x, Gibco). CCL-206 mouse lung fibroblasts ([MLg2908, CCL206], ATCC, Wesel, Germany) were cultured in DMEM/F12 medium supplemented with 10% FBS, penicillin/streptomycin (100 U/mL), glutamine (1%) and Amphotericin B. Cells were incubated at 37°C, 5% CO_2_ humidified environment. MRC5 or CCL206 fibroblasts were starved once grown to 80% confluence in the 6-well culture plates. The starvation medium contains the same components as culture medium described above, but with only 0.5% FBS. After 24 h starvation, cells were then incubated with either vehicle, TGF-β1 and/or investigational compounds (Table 1) in serum deprivation medium for 48 h. MRC5 fibroblasts were collected for gene expression analysis. CCL206 fibroblasts were washed three times with warm PBS and proliferation-inactivated by incubation in mitomycin C (10 μg/ml, Sigma #M4287) for 2 h, followed by three washes in warm PBS and trypsinization prior to mixing with epithelial cells, as described previously (Ng-Blichfeldt et al., 2018; Ng-Blichfeldt et al., 2019).

### Mouse Epithelial Cell Isolation

Epithelial (EpCAM^+^) cells were isolated from lungs of 8–12 week old male and female C57Bl6 mice with microbeads as described previously (Ng-Blichfeldt et al., 2018; Ng-Blichfeldt et al., 2019; Wu et al., 2019a). Lungs of mice were flushed through the heart with PBS, instilled with dispase (BD Biosciences, Oxford, United Kingdom, #354235) and low-melt agarose (Sigma Aldrich, Poole, United Kingdom #A9414), and incubated at room temperature (RT) for 45 min. Trachea and extrapulmonary airways were removed, and the remaining lobes were homogenized in DMEM medium with dnase1 (Applichem, Germany #A3778). The resulting suspension was passed through a cell strainer with the size of 100 μm, incubated with microbeads conjugated to antibodies for CD45 (Miltenyi Biotec, Teterow, Germany #130–052–301) and CD31 (Miltenyi, #130–097–418), and passed through LS columns (Miltenyi #130–091–051). The CD31^-^/CD45^-^ suspension was then enriched for epithelial cells by positive selection using EpCAM (CD326) microbeads (Miltenyi #130–105–958). EpCAM^+^ cells were resuspended in DMEM with 10% FBS.

### Organoid Culture

The organoid assay was established as described previously (Ng-Blichfeldt et al., 2018; Ng-Blichfeldt et al., 2019; Wu et al., 2019a). EpCAM^+^ cells were combined with fibroblasts at a 1:1 ratio in DMEM/F12 (10% FBS) at a density of 2 * 10^5^ cells/ml. The cell suspension was then diluted 1:1 (v/v) with Matrigel (Fisher Scientific, Landsmeer, The Netherlands) and were seeded into transwell inserts (Thermo Fischer Scientific, Waltham, United States #10421761) witin 24-well plates (100 µl/insert). Cultures were maintained in DMEM/F12 with 5% (v/v) FBS, 2 mM glutamine, antibiotics, insulin-transferrin-selenium (1x, Gibco #15290018), recombinant mouse EGF (0.025 μg/ml, Sigma #SRP3196), bovine pituitary extract (30 μg/ml, Sigma #P1476), and freshly added all-trans retinoic acid (0.01μM, Sigma #R2625) at 37°C with 5% CO2. Media was refreshed every 2–3 days. The total number of organoids per well was counted manually 14 days after seeding using a light microscope at ×20 magnification. Organoid diameter was measured at the same day using a light microscope connected to NIS-Elements software. Thereafter, organoid cultures were fixed for immunofluorescence.

### Gene Expression Analysis

Total RNA was extracted from PCLS by automated purification using the Maxwell 16 instrument and the corresponding Maxwell 16 LEV simply RNA tissue kit (Promega, Madison, United States) according to the manufacturer’s instructions. Total RNA was extracted form MRC5 fibroblasts using the TRIzol method. RNA concentrations were determined using a ND-1000 spectrophotometer and equal amounts of total mRNA were then reverse transcribed (Promega, Madison, United States 0). The cDNA was subjected to real-time qPCR (Westburg, Leusden, The Netherlands) using SYBR green as the DNA binding dye (Roche Applied Science, Mannheim, Germany) on an Illumina Eco Real-Time PCR system (Westburg, Leusden, the Netherlands), with denaturation at 94°C for 30 s, annealing at 59°C for 30 s and extension at 72°C for 30 s for 40 cycles followed by 10 min at 72°C. Real-time qPCR data were analyzed using LinRegPCR analysis software and the amount of target gene was normalized to the endogenous reference gene 18S ribosomal RNA for mouse PCLS and to SDHA for human fibroblasts. The specific forward and reverse primers used are listed in Supplementary Table S1.

### Immunofluorescence

MRC5 fibroblasts were cultured on the coverslips within the culture plate to perform immunofluorescence experiments. Cells were washed twice with PBS and fixed with 4% paraformaldehyde (PFA) for 10 min at RT. Then cells were washed again twice with PBS and were incubated with Alxea Fluor™ 488 Phalloidin (ThermoFisher, A12379) 1:40 diluted in PBS for 20 min at RT. After washing three times with PBS, the coverslips were transferred onto glass slides and were mounted by mounting medium contains DAPI (Abcam #ab104139).

Organoid were fixed within ice-cold acetone/methanol (1:1) medium for 15 min at −20°C, then were blocked in PBS, supplemented with 5% BSA (Ng-Blichfeldt et al., 2018; Ng-Blichfeldt et al., 2019; Wu et al., 2019a). Cultures were incubated with primary antibodies Rb anti-pro-surfactant protein C (pro-SPC, Millipore AB3786) and mouse anti-acetylated tubulin (ACT, Santa Cruz sc-23950) diluted 1:200 in PBS with 0.1% BSA and 0.1% Triton-X100 at 4°C overnight. Thereafter, cultures were washed 3 times in PBS (>1 h between washes) and incubated with secondary antibodies donkey anti-rabbit (Jackson Immunoresearch, 711–165–152) and donkey anti-mouse (Jackson Immunoresearch, 711–165–152) diluted 1:200 at room temperature for 2.5 h. Cultures were excised from inserts and mounted on glass slides with mounting media containing DAPI (Abcam #ab104139) and glass coverslips.

Immunofluorescence was visualized using a Leica SP8 confocal microscope (Wetzlar, Germany), and images obtained with Leica LAS software.

### Data Analysis

Statistical evaluation of differences was performed using one-way ANOVA followed by a Student-Newman Keuls post-hoc test. Differences were considered to be statistically significant when *p* < 0.05. GraphPad Prism eight software was used to perform statistical analysis.

## Results

### Novel ROCK Inhibitors and Their Kinase Selectivity

**FIGURE 1:**
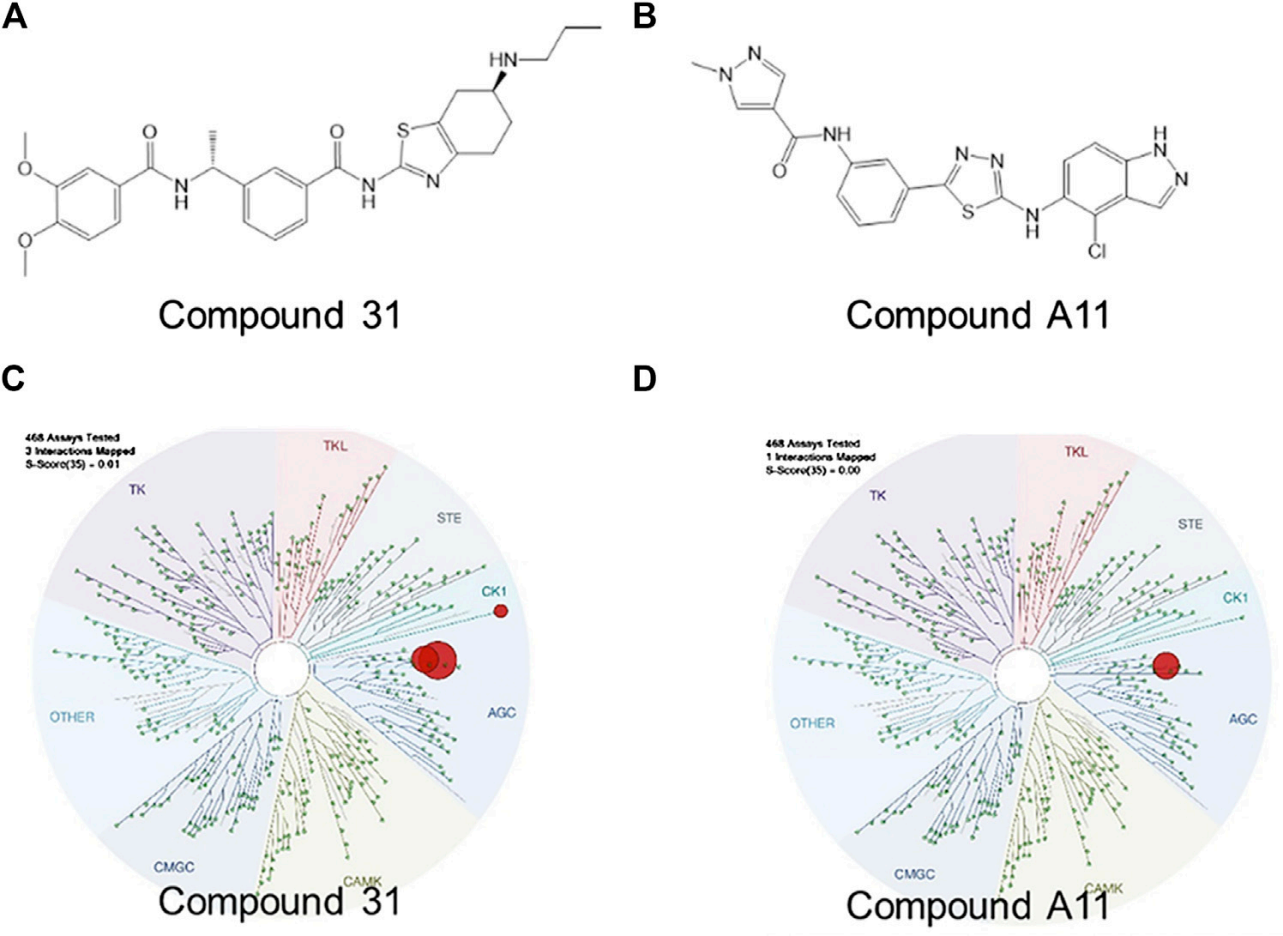
Pharmacological characterization of the ROCK inhibitors used. **(A-B)**, The molecular structure of compound 31 and compound A11 respectively. **(C-D)**, The kinase activity of compound 31 and A11 respectively.

Over the past decades, numerous ROCK inhibitors have been developed from a variety of distinct scaffolds, however, few examples of selective ROCK2 inhibitors have been described. We selected two potent ROCK inhibitors (ROCKi) spotted in public patents, one is a dual ROCK1 and 2 (ROCK1/2) inhibitor naming compound 31 (example 2 of WO 2012/006202); and the other is a ROCK2 selective inhibitor naming compound A11 (example A11 of WO 2016/138335). The enzymatic potency of these two compounds is shown in Table 1. Compound 31 and compound A11 were screened in a competitive assay against a large panel (>400) of human kinases (Kinome Scan®, Discoverx) at the concentration of 100 nM, >10-fold higher than the enzymatic IC_50_ against ROCK2. The graphical view of kinome scan is reported in Figure1 where only interactions under the threshold for residual activity of 35% are displayed and potentially indicating off-target interactions. Compound 31 shows three spots, two related to ROCKs (ROCK1: 0% CTRL and ROCK2: 0% CTRL) and only one off-target interaction at 9.3% related to VRK2. Compound A11 showed only interaction with ROCK2 at 0.35% vs CTRL.

### Effects of Dual ROCK1/2 and ROCK2 Selective Inhibition on Transforming Growth Factor-β-Induced Myofibroblast Differentiation

**FIGURE 2:**
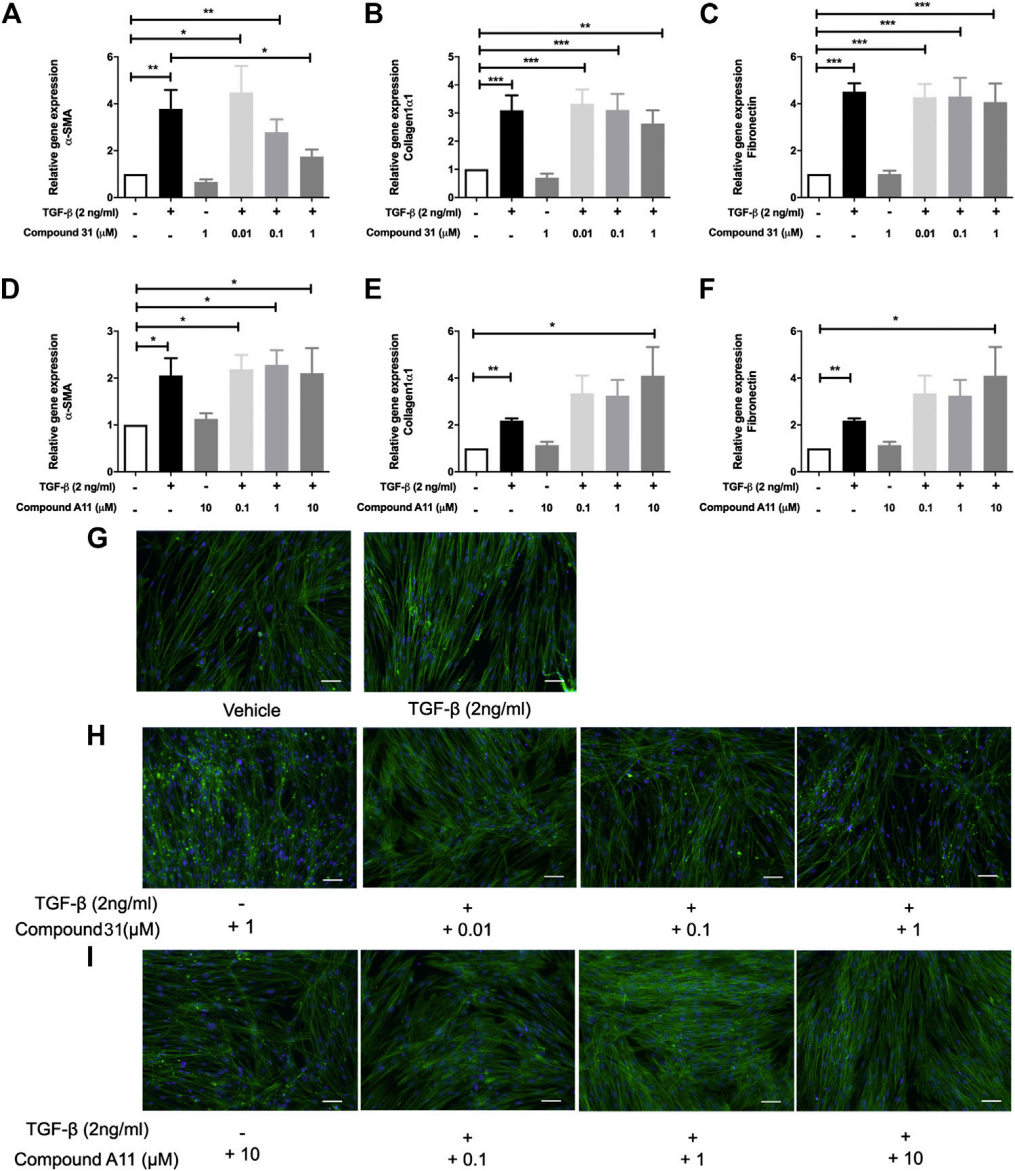
Effects of dual ROCK1/2 vs ROCK2 selective inhibition on TGF-β induced myofibroblast differentiation of human lung fibroblasts. **(A–C)**, mRNA expression of α-sm-actin (α-SMA), collagen 1α1, and fibronectin (FN) in MRC5 cells treated with TGF‐β (0-, 2 ng/mL) + compound 31 (0.01 μM, 0.1 μM and 1 μM). **(D–F)**, mRNA expression of α-sm-actin (α-SMA), collagen 1α1, and fibronectin (FN) in MRC5 cells treated with TGF‐β (0‐, 2 ng/mL) ± compound A11 (0.1 μM, 1 μM and 10 μM). **(G)**, Representative phalloidin staining of MRC5 cells treated with TGF-β (0‐, 2 ng/mL). **(H)**, Representative phalloidin staining of MRC5 fibroblasts cells treated with TGF-β (0‐, 2 ng/mL) + compound 31 (0‐, 0.01‐, 0.1‐, 1‐ μM). **(I)**, Representative phalloidin staining of MRC5 cells treated with TGF‐β (0‐, 2 ng/mL) ± compound A11 (0‐, 0.1‐, 1‐, 10‐ μM). Blue: dapi; green: F‐actin. Scale bar = 100 μm.

To induce myofibroblast differentiation, MRC5 human lung fibroblasts were treated with TGF-β1 (2 ng/ml) for 48 h. TGF-β1 increased the mRNA levels of α -smooth muscle actin (α-SMA), collagen 1α1 (Col1α1) and fibronectin (FN) significantly (Figure 2). To investigate the effect of ROCK inhibition on TGF-β driven airway remodeling, compound 31 (0.01-, 0.1-, and 1 μM) and compound A11 (0.1-, 1-, and 10 μM) were applied to the fibroblasts treated with TGF-β. Compound 31 had no effect on mRNA expression of FN in response to TGF-β, but significantly decreased the α-SMA gene expression level and tended to decrease the Col1α1 expression level in a concentration dependent manner (Figures 2A–C). In contrast, the ROCK2 selective compound A11 was not able to alter the expression of α-SMA, and if anything, tended to increase the expression of Col1α1 and FN in combination with TGF-β (Figure 2D–F). Neither compound 31 (1 μM) nor compound A11 (10 μM) had an effect on its own (i.e. in the absence of TGF-β). Next, we stained the MRC5 fibroblasts with phalloidin, which is able to bind and stabilize the filamentous actin (F-actin). As shown in Figure 2G, TGF-β treatment gave more F-actin stress fibers than the vehicle control, whereas treatment with compound 31 reduced stress fiber formation in combination with TGF-β (Figure 2H). Compound A11 on the other hand had no inhibitory effect and if anything, tended to enhance the formation of stress fibers (Figure 2I), similar to the previous findings on the gene expression of α-SMA.

**FIGURE 3:**
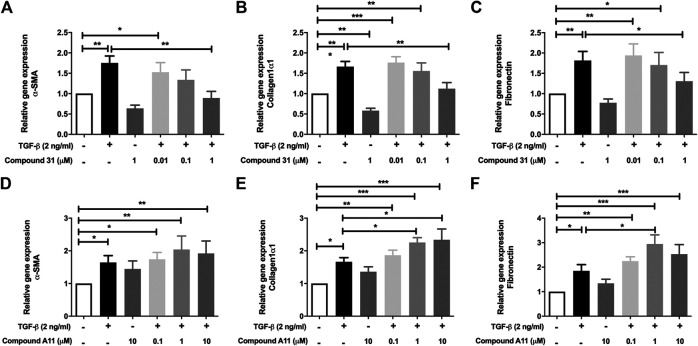
Effects of dual ROCK1/2 vs ROCK2 selective inhibition on TGF-β induced myofibroblast differentiation in murine PCLS. **(A–C)**, mRNA expression of α-sm-actin (α-SMA), collagen 1α1, and fibronectin (FN) in murine PCLS treated with TGF-β (0-, 2 ng/mL) ± compound 31 (0.01 μM, 0.1 μM and 1 M). **(D–F)**, mRNA expression of α-smooth-actin (α-SMA), collagen 1α1, and fibronectin (FN) in murine PCLS treated with TGF-β (0-, 2 ng/mL) ± compound A11 (0.1 μM, 1 μM and 10 μM).

In the murine PCLS, the mRNA levels of α-SMA, Col1α1 and FN were significantly increased by TGF-β treatment (Figure 3). In this model system, the increased mRNA expression of α-SMA, Col1α1 and FN were all significantly reduced by compound 31 in a concentration-dependent manner (Figures 3A–C). Interestingly, in line with the fibroblast data, compound A11 had no effect on the increased level of α-SMA, but significantly enhanced levels of Col1α1 and FN in the presence of TGF-β (Figures 3D–F). Taken together, these results indicate that dual ROCK 1/2 inhibition but not ROCK 2 selective inhibition is able to reduce the increased mRNA level of α-SMA, Col1α1 and FN in response to TGF-β. Unfortunately, the already high background expression of α-SMA, collagen 1 and fibronectin in the lung slice prevented us from being able to pick up strong enough effects of TGF-β at the protein level with semi-quantitative methods such as western blot and immunofluorescence microscopy (not shown), supporting the idea that the lung slice is suitable to pick up early changes, but not later stage changes associated with TGF-β induced fibrosis (Kasper et al., 2004).

### Effects of Dual ROCK1/2 and ROCK2 Selective Inhibition on Transforming Growth Factor-β Induced Alterations in Alveolar Epithelial Organoid Formation

**FIGURE 4:**
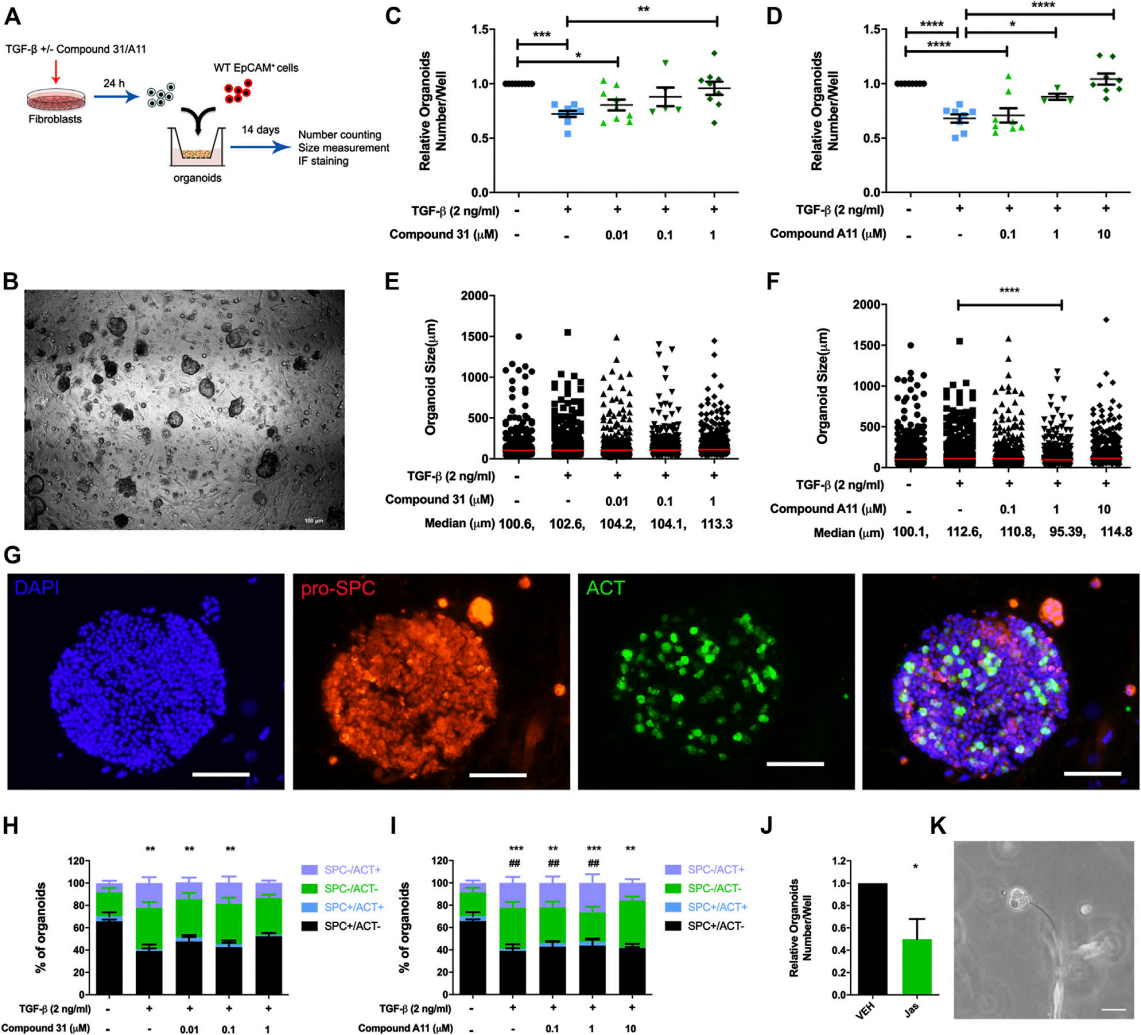
ROCK inhibition restored the reduction of organoid number. **(A)**, The schematic of the organoid experimental setup. **(B)**, Representative images of epithelial organoids obtained, scale bar = 100 μm. **(C-D)**, Total organoid number at day 14 after co-culture of mouse CD31-/CD45-/EpCam+ cells with CCL206 lung fibroblasts pretreated with TGF-β (0‐, 2 ng/mL) ± compound 31 (0.01 μM, 0.1 μM and 1 μM; panel C) or ompound A11 (0.1 μM, 1 μM and 10 μM; panel D), N = 4 - 6, mean ± SEM is shown, *p < 0.05, **p < 0.01. **(E-F)**, Organoid size measured at day 14 for the same experimental conditions as shown in C-D. **(G)**, Representative images of immunofluorescence staining of organoids, blue: dapi, red: pro-spc, green: acetylated tubulin (ACT), the scale bar = 100 μm. **(H-I)**, Quantification of organoid proportion expressing pro-proSPC+ or ACT+ at day 14. *p < 0.05, **p < 0.01, ***p < 0.001 compared to vehicle for proSPC+/ACT- alveolar organoids, #; *p* < 0.05, #*p* < 0.01 compared to vehicle for proSPC-/ACT+ airway organoids. **(J)**, Total organoid number at day14 of mouse CD31-/CD45-/EpCam+ cells with CCL206 lung fibroblasts pretreated with with jasplakinolide (100 nM). **(K)**, Image of a fibroblast cell touching an early formed organoid from day-3, scale bar = 100 μm.

Previous studies from our group demonstrated that TGF-β activation impairs the fibroblast ability to support adult lung epithelial progenitor cells to form organoids (Ng-Blichfeldt et al., 2019). To investigate whether the novel ROCK inhibitors are able to restore the defective organoid formation, we designed the organoid assay as shown in Figure 4A. Murine CCL206 lung fibroblasts were differentiated into myofibroblasts by TGF-β (2 ng/ml) in the absence or presence of compound 31/compound A11 for 24 h. Afterward, the pretreated fibroblasts were extensively washed to remove the stimuli and co-cultured with freshly isolated mouse lung epithelial cells (CD31^-^/CD45^-^/EpCAM^+^ cells). Myofibroblast differentiation with TGF-β reduced the number of epithelial organoids formed at day 14, in line with our previous findings (12). Both compound 31 and compound A11 were able to restore the reduced numbers of organoids in a concentration-dependent manner to levels seen in the control cultures (Figures 4C, D). TGF-β induced myofibroblast differentiation had no impact on the median diameter of the organoids formed (Figures 4E, F). In combination with compound A11, however, the median diameter was slightly decreased by 1 µM yet increased by 10 µM compound compared to TGF-β stimulation alone (Figure 4F). Myofibroblast differentiation with TGF-β significantly reduced the proportion of alveolar (proSPC^+^/ACT^−^) organoids yet increased the proportion of airway organoids (proSPC^−^/ACT^+^) quantified after immunofluorescence staining (Figures 4G–I). This was partially restored by dual ROCK1/2 inhibition with compound 31, whereas ROCK2 selective inhibition by compound A11 had no such effect (Figures 4H, I).

As the main effect of ROCK inhibition on the fibroblast was to reduce α-sm-actin expression and stress fiber formation (Figure 2), we also investigated the role of stress fiber formation in itself on the ability of fibroblasts to support organoid formation. Pretreatment of fibroblasts with jasplakinolide, a compound that restricts mobility by promoting actin polymerization, appeared sufficient to mimic the effect of TGF-β (Figure 4J). Interestingly, we spotted direct cell-cell contact between fibroblasts and developing epithelial organoids in the assay (Figure 4K). This is consistent with our observation that in our lung organoid cultures, direct contact of fibroblasts and alveolar epithelial cells is essential for organoid growth. Thus, organoids only form if fibroblasts and epithelial progenitors are in direct contact within the Matrigel. Organoids do not form if fibroblasts are cultured on the adjacent bottom chamber or if conditioned media of fibroblasts is used, confirming the essential role of fibroblasts in alveolar organoid formation (Supplementary Figure S1).

### ROCK Inhibition Effects Secreted Factors From Fibroblasts That Support Organoid Formation

**FIGURE 5:**
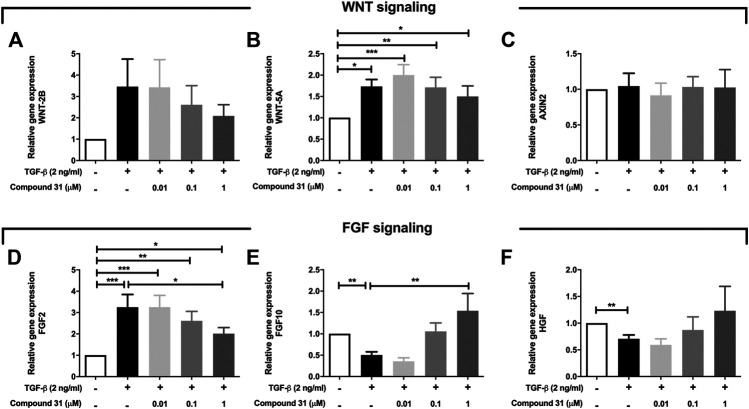
Expression of WNT signaling and FGF signaling pathway genes in response to TGF-β and compound 31. **(A-F)**, The mRNA expression of WNT-2B, WNT-5A, AXIN2, FGF2, FGF10 and HGF in MRC5 cells treated with TGF-β (0-, 2 ng/mL) ± compound 31 (0.01 μM, 0.1 μM and 1 μM). *p < 0.05, **p < 0.01, ***p < 0.001.

Fibroblasts secrete several growth factors that are essential to alveolar organoid formation, which is skewed by TGF-β treatment (Ng-Blichfeldt et al., 2019). Thus, we investigated whether ROCK inhibition may impact on the expression of these secreted factors. We focused these studies on compound 31 as this compound had the strongest impact on reversing the TGF-β effects throughout this study. We examined the mRNA expression level of several key components of WNT signaling and FGF signaling pathways, which play an important role in tissue regeneration. Intriguingly, TGF-β increased the mRNA level of WNT-5A (*p* < 0.05) and WNT-2B in MRC5 fibroblasts but had no influence on AXIN2 (Figures 5A–C) expression. When compound 31 was added, the increased expression of those WNT ligands was normalized in a concentration-dependent manner (Figures 5A–C). Moreover, TGF-β stimulation increased FGF2 mRNA expression and decreased FGF-7 and HGF expression (Figures 5D–F). Compound 31 reduced FGF2 expression and restored FGF7 and HGF expression in a concentration-dependent manner (Figure 5D–F). Thus, TGF-β activation in fibroblasts distorts the mesenchymal-epithelial interactions via WNT signaling and FGF signaling pathways, which was reversed by dual ROCK 1/2 inhibition.

## Discussion

A better understanding of the mechanisms that regulate phenotype and function of lung (myo)fibroblasts may lead to the identification of therapeutic targets. TGF-β is a master regulator of myofibroblast differentiation in fibrosis, as evident from several *in vitro* and *in vivo* studies (Meng et al., 2016; Nagai et al., 2019; Ng-Blichfeldt et al., 2019; Noe et al., 2019; Saidi et al., 2019). In this study, we investigated the potential of two novel ROCK inhibitors with different selectivity against two ROCK isoforms in counteracting TGF-β induced effects on myofibroblast differentiation and alveolar epithelial progenitor organoid formation. Interactions between pulmonary fibroblasts and epithelial cells not only contributes to homeostasis but also to lung repair in many pathological conditions (Demayo et al., 2002; Horowitz and Thannickal, 2006; Hirota and Martin, 2013; El Agha et al., 2017; Wu et al., 2019a; Ng-Blichfeldt et al., 2019; Noe et al., 2019; Zepp and Morrisey, 2019). We previously showed (Ng-Blichfeldt et al., 2019) that this interaction is disturbed if fibroblasts are transdifferentiated into myofibroblasts by TGF-β. A reduction in SPC^+^ alveolar organoids was observed after TGF-β treatment and an increase in the number of ACT^+^ airway organoids. ROCK 1 and ROCK 2 inhibition was able to restore the reduced organoid formation in response to TGF-β in current study and dual inhibition partially restored the number of SPC^+^ alveolar organoids, whereas selective ROCK2 inhibition did not. We propose two mechanistic explanations for this TGF-β effect, being restriction of fibroblast motility and alterations in secreted factors, both of which are normalized by dual ROCK1/2 inhibition. Indeed, fibroblasts are needed in co-culture with epithelial cells in order to form organoids and organoids form only if fibroblasts are in direct cell-cell contact. We speculated that fibroblast motility is required for this effect. TGF-β restricts fibroblast motility by increasing α-sm-actin stress fibers. In support, we show that dual ROCK1/2 inhibition can inhibit both the stress fiber formation and the reduced organoid numbers in response to TGF-β. Furthermore, pretreatment of fibroblasts with jasplakinolide, which restricts fibroblast mobility by inducing α-sm-actin stress fiber formation, is sufficient to disturb the organoid formation to a similar extent as TGF-β (Ng-Blichfeldt et al., 2019).

In the past 2 decades, the development of pharmacological ROCK inhibitors has gained increasing interest; however, in the majority of published studies classic ROCK inhibitors, such as Y27632 and (hydroxy) fasudil, both of which target the ATP-dependent kinase domain of ROCK1 and ROCK2, are utilized. The two ROCK isoforms, ROCK1 and ROCK2, are structurally similar sharing ∼60% overall amino acid identity, and within the N-terminal kinase domain, they are ∼90% homologous. Accordingly, the design of isoform selective inhibitors has until now been very challenging. Unfortunately, both these first generation ROCK inhibitors have poor ROCK inhibition potency, and are additionally unselective against a range of other kinases, especially those in the AGC family (Tumbarello and Turner, 2006; Hallgren et al., 2012; Pireddu et al., 2012; Rath and Olson, 2012; Vigil et al., 2012; Xueyang et al., 2016; Huang et al., 2018; Cantoni et al., 2019). To fill this gap, we selected, as tool compounds, two ROCK inhibitors previously described in two distinct patents, a ROCK 1 and 2 inhibitor (compound 31), and a ROCK2 selective inhibitor (compound A11). Our results show that they elicit non-identical effects in TGF-β-induced remodeling. We show an increase of α-SMA expression in TGF-β activated human fibroblasts and the murine PCLS, and only compound 31 was able to downregulate the contractile marker expression in both models, indicating that dual ROCK 1/2 inhibition is necessary for preventing contractile activity in pulmonary fibroblasts. A recent study (Knipe et al., 2018) using genetic ROCK inhibition showed that there is no significant decrease in α-SMA expression with individual ROCK1 or ROCK2 knockdown as compared with nontargeting siRNA in response to TGF-β, however, they showed a reduction of α-SMA expression when ROCK1 and ROCK2 were simultaneously knocked down. Together, this indicates that dual ROCK1 and ROCK2 inhibition profoundly attenuates the contractility of fibroblasts. Interestingly, we found dual ROCK1 and ROCK2 inhibition prevented the synthesis of collagen expression induced by TGF-β, however, ROCK2 inhibition tended to enhance it, suggesting the activity of each ROCK isoform may play counteractive roles in response to TGF-β signaling. Knipe R.S., et al., also observed a reduction in collagen expression with the knockdown of ROCK1 or with both isoforms in response to TGF-β (Knipe et al., 2018). Additionally, Yu Zhang, et al., showed that ROCK2-siRNA on TGF-β-stimulated ARE luciferase reporter expression was blocked by co-expression of ROCK2; and the inhibition of human ROCK2 overexpression in response to TGF-β was blocked in the presence of ROCK kinase inhibitor Y27632 in human liver cells, suggesting that ROCK2 acts as a negative regulator of the TGF-β signaling pathway (Zhang et al., 2009).

Rho kinases may regulate multiple signaling pathways via different substrates. In addition, ROCK inhibition is presumably playing a major role in regulating secreted factors. We examined several key components of the WNT signaling and FGF signaling pathway, which are known to contribute to epithelial development and regeneration (KathernMyrna et al., 2012; Ornitz and Itoh, 2015; Dean and Lloyd, 2017; Kim et al., 2018; Prince, 2018; Puschhof and Clevers, 2018; Villar et al., 2019). Increasing evidence demonstrated aberrant WNT signaling results in fibrotic lung diseases (Baarsma and Königshoff, 2017; Cao et al., 2018; Martin-Medina et al., 2018). Vuga et al. (2009) showed enhanced WNT-5A signaling that contributes to ECM deposition, suggesting ROCK inhibition may repress the ECM deposition via WNT signaling (Vuga et al., 2009). Our results show that the non-canonical WNT ligand WNT-5A was significantly increased in response to TGF-β, whereas this was normalized by compound 31. This is consistent with our previous findings (Wu et al., 2019a) showing that the mesenchymal WNT-5A signaling represses alveolar epithelial progenitor growth, and suggests that pharmacological inhibition of ROCK1/2 in fibroblasts may help to promote canonical WNT signaling in lung repair.

Furthermore, functional alterations in the FGF signaling pathway were observed. Fibroblast growth factors (FGFs) are members of the heparin-binding growth factor family that are often involved in morphogenesis and wound repair and FGF signaling dysregulations is implicated in many disorders (Ornitz and Itoh, 2015; Fehrenbach et al., 2017; Ohgiya et al., 2017; Plikus et al., 2017; Shiraishi et al., 2019; Weiner et al., 2019). FGF2 has attracted increasing attention in lung biology recently and is reported as an important factor in airway remodeling by increasing the deposition of proteoglycans resulting in bronchial hyperresponsiveness in asthmatic airways (Kim et al., 2018). FGF10, a member of the FGF7-subfamily, is widely reported as a primary regulator for branching morphogenesis, cellular differentiation, and response to injury (Prince, 2018; Weiner et al., 2019; Zepp and Morrisey, 2019). We reported previously (Ng-Blichfeldt et al., 2018, 2019) that mesenchymal FGF7, HGF, and FGF10 support alveolar organoid growth, and exogenous FGF7 and HGF rescue TGF-β-induced reduction in organoid number when added to the organoid culture. Our transcriptional analysis also showed that TGF-β upregulated the expression of FGF2 but downregulated the expression of FGF10 and HGF in human fibroblasts and compound 31 counteracts TGF-β effects. Additionally, a recent publication showed that the gene expression of Col1α1, FN1, FGF2 and HGF were all increased in lungs of the patients who died from Covid-19 (Ackermann et al., 2020), genes that are all TGF-β responsive, yet inhibited by dual ROCK1/2 as we show in this study. Taken together, these results suggest that TGF-β elicits modifications of contractility and secreted factors in fibroblasts, and ROCK 1 and 2 inhibition is able to counteract such effects.

According to the human lung cell atlas (https://asthma.cellgeni.sanger.ac.uk/) (Schiller et al., 2019) and the IPF lung cell atlas (http://www.ipfcellatlas.com/) (Adams et al., 2019), the expression of ROCK1 is much higher than ROCK2 in both pulmonary epithelial cells and stromal cells (Supplementary Figure S2). Interestingly, ROCK1 increased in (myo)fibroblasts in response to IPF pathology, however, ROCK2 shows opposite alterations in fibroblasts and myofibroblasts (Supplementary Figure S3). These data suggest that the role of ROCK1 in IPF pathology (at least at transcriptomic level) might be more profound as compared to that of ROCK2 and might explain why the protective effect of dual ROCK1/2 inhibition is more profound than ROCK2 selective inhibition in the current study. In further support of this contention, the level of ROCK1 has been demonstrated to function as a clinical progression marker for IPF (Park et al., 2014; Knipe et al., 2016). This is consistent with our functional studies showing that ROCK2 selective inhibition is less effective than ROCK1/2 inhibition. An earlier study (Hallgren et al., 2012) showed that fibroblasts isolated from the parenchyma of severe COPD patients that have more contractile phenotypes are associated with enhanced ROCK1 expression, and the ROCK inhibitor Y27632 blocked this contraction. Thus, it would be interesting to evaluate the effect of selective pharmacological inhibition of ROCK1 in future studies; unfortunately, ROCK1 selective inhibitors are currently not available. Giving the structural similarity between ROCK1 and ROCK2, there are no immediate structural features that can be exploited to design ROCK1 selective inhibitors, while in case of ROCK2, the optimization of van der Waals contacts between the more flexible glycine rich loop and the portion of the molecule underneath P-loop can favor selectivity. However, it is hard to use these observations to guide the design of a ROCK1 selective inhibitor. In addition, hypotensive effects of systemic ROCK inhibition appear to be associated mainly with ROCK1 and this may have driven for discovery of ROCK2 selective inhibitors as safer drugs. Since ROCK1 isoform-selective inhibitors are not currently available, additional studies using genetic approach to specifically delete ROCK1 or ROCK2 will be necessary to elucidate specific roles of these two isoforms in the development of pulmonary remodeling and repair. Such studies will be needed to determine whether an inhibitor for either ROCK1 or ROCK2 may be as effective as a nonselective inhibitor and may be better tolerated.

In conclusion, pharmacological inhibition of both ROCK 1 and 2 isoforms effectively prevents TGF-β-induced fibroblast myofibroblast differentiation and counteracts TGF-β induced growth inhibition of alveolar epithelial progenitors. Selective ROCK2 inhibition does not affect TGF-β effects on extracellular matrix and contractile proteins but does reverse the TGF-β induced inhibition of organoid growth. Our results indicate that mesenchymal ROCK1/2 inhibition may be a potential therapeutic target to promote lung repair.

## Data Availability Statement

The original contributions presented in the study are included in the article/Supplementary Material, further inquiries can be directed to the corresponding author.

## Ethics Statement

The animal study was reviewed and approved by University of Groningen Committee for Animal Experimentation (license numbers: AVD10500201581 and AVD105002015303).

## Author Contributions

LEMK, and RG designed and supervised the project; XW, VV, MEW, J-P-N-B, and AM performed the experiments. LEMK, RG, XW, VV, MEW, analyzed data; LEMK, RG, XW, GV, AA, and FF, interpreted results or experiments; XW, GV, AA, and FF, prepared the figures; XW, LEMK, and FF, drafted manuscript; all authors edited and revised manuscript; LEMK, RG, XW, VV, MEW, J-P-N-B, GV, AA, FF, and AM, approved final version of manuscript.

## Funding

The authors declare that this study received funding from Chiesi Farmaceutici S.p.A., the Lung Foundation Netherlands (5.1.17.166), NWO (40-00506-98-9021 and 175-010-2009-023) and China Scholarship Council (CSC, 201707720065). Author G.V., A.A. and F.F. were employed by the funder Chiesi. The funder had the following involvement with the study: The funder was involved in the study design, interpretation of data, the writing of this article and the decision to submit it for publication.

## Conflict of Interest

Author GV, AA and FF are employees of Chiesi Farmaceutici SpA. Author LK and RG have received funding for research and lecture fees from Boehringer Ingelheim. Author MW, VV and LK were employed by the company Aquilo BV.

The remaining authors declare that the research was conducted in the absence of any commercial or financial relationships that could be construed as a potential conflict of interest.

## References

[B1] Ackermann M. Verleden S. E. Kuehnel M. Haverich A. Welte T. Laenger F. (2020). Pulmonary vascular endothelialitis, thrombosis, and angiogenesis in covid-19. N. Engl. J. Med., 383, 120–128. 10.1056/NEJMoa2015432 32437596 PMC7412750

[B2] Adams T. S. Schupp J. C. Poli S. Ayaub E. A. Neumark N. Ahangari F. (2019). Single Cell RNA-seq reveals ectopic and aberrant lung resident cell populations in idiopathic pulmonary fibrosis. bioRxiv. 10.1101/759902 10.1126/sciadv.aba1983 PMC7439502 32832599

[B3] Baarsma H. A. Königshoff M. (2017). “WNT-er is coming”: WNT signalling in chronic lung diseases. Thorax. 72 (8), 746–759. 10.1136/thoraxjnl-2016-209753 28416592 PMC5537530

[B4] Cantoni S. Cavalli S. Pastore F. Accetta A. Pala D. Vaccaro F. (2019). Pharmacological characterization of a highly selective Rho kinase (ROCK) inhibitor and its therapeutic effects in experimental pulmonary hypertension. Eur. J. Pharmacol. 850, 126–134. 10.1016/j.ejphar.2019.02.009 30753868

[B5] Cao H. Wang C. Chen X. Hou J. Xiang Z. Shen Y. (2018). Inhibition of Wnt/β-catenin signaling suppresses myofibroblast differentiation of lung resident mesenchymal stem cells and pulmonary fibrosis. Sci. Rep. 8, 1–14. 10.1038/s41598-018-28968-9 30206265 PMC6134002

[B6] Dean C. H. Lloyd C. M. (2017). Lung alveolar repair: not all cells are equal. Trends Mol. Med. 23, 871–873. 10.1016/j.molmed.2017.08.009 28870601

[B7] Demayo F. Minoo P. Plopper C. G. Schuger L. Shannon J. Torday J. S. (2002). Mesenchymal-epithelial interactions in lung development and repair: are modeling and remodeling the same process? Am. J. Physiol. Lung Cell Mol. Physiol. 283 (3), L510–L517. 10.1152/ajplung.00144.2002 12169568

[B8] dos Santos T. M. Righetti R. F. Camargo L. d. N. Saraiva-Romanholo B. M. Aristoteles L. R. C. R. B. de Souza F. C. R. (2018). Effect of anti-IL17 antibody treatment alone and in combination with Rho-kinase inhibitor in a murine model of asthma. Front. Physiol. 9, 1–19. 10.3389/fphys.2018.01183 30233389 PMC6134017

[B9] El Agha E. Kramann R. Schneider R. K. Li X. Seeger W. Humphreys B. D. (2017). Mesenchymal stem cells in fibrotic disease. Cell Stem Cell. 21, 166–177. 10.1016/j.stem.2017.07.011 28777943

[B10] Fehrenbach H. Wagner C. Wegmann M. (2017). Airway remodeling in asthma: what really matters. Cell Tissue Res. 367, 551–569. 10.1007/s00441-016-2566-8 28190087 PMC5320023

[B11] Florian J. Watte G. Teixeira P. J. Z. Altmayer S. Schio S. M. Sanchez L. B. (2019). Pulmonary rehabilitation improves survival in patients with idiopathic pulmonary fibrosis undergoing lung transplantation. Sci. Rep. 9, 1–6. 10.1038/s41598-019-45828-2 31249363 PMC6597536

[B12] Grzela K. Litwiniuk M. Zagorska W. Grzela T. (2016). Airway remodeling in chronic obstructive pulmonary disease and asthma: the role of matrix metalloproteinase-9. Arch. Immunol. Ther. Exp. 64, 47–55. 10.1007/s00005-015-0345-y PMC4713715 26123447

[B13] Hallgren O. Rolandsson S. Andersson-Sjöland A. Nihlberg K. Wieslander E. Kvist-Reimer M. (2012). Enhanced ROCK1 dependent contractility in fibroblast from chronic obstructive pulmonary disease patients. J. Transl. Med. 10, 1–11. 10.1186/1479-5876-10-171 22913419 PMC3477051

[B14] Hirota N. Martin J. G. (2013). Mechanisms of airway remodeling. Chest. 144, 1026–1032. 10.1378/chest.12-3073 24008953

[B15] Horowitz J. C. Thannickal V. J. (2006). Epithelial-mesenchymal interactions in pulmonary fibrosis. Semin. Respir. Crit. Care Med. 27 (6), 600–612. 10.1055/s-2006-957332 17195137 PMC2225581

[B16] Htwe S. S. Cha B. H. Yue K. Khademhosseini A. Knox A. J. Ghaemmaghami A. M. (2017). Role of Rho-Associated coiled-coil forming kinase isoforms in regulation of stiffness-induced myofibroblast differentiation in lung fibrosis. Am. J. Respir. Cell Mol. Biol. 56, 772–783. 10.1165/rcmb.2016-0306OC 28225294

[B17] Huang L. Dai F. Tang L. Bao X. Liu Z. Huang C. (2018). Distinct roles for ROCK1 and ROCK2 in the regulation of oxldl-mediated endothelial dysfunction. Cell. Physiol. Biochem. 49, 565–577. 10.1159/000492994 30165352

[B18] Jiang C. Huang H. Liu J. Wang Y. Lu Z. Xu Z. (2012). Fasudil, a Rho-kinase inhibitor, attenuates bleomycin-induced pulmonary fibrosis in mice. Int. J. Mol. Sci. 13 (7), 8293–8307. 10.3390/ijms13078293 22942703 PMC3430234

[B19] Kasper M. Seidel D. Knels L. Morishima N. Neisser A. Bramke S. (2004). Early signs of lung fibrosis after *in vitro* treatment of rat lung slices with CdCl2 and TGF-β1. Histochem. Cell Biol. 121 (2), 131–140. 10.1007/s00418-003-0612-6 14752665

[B20] KathernMyrna E. D. V. M. SimonPot A. D. V. M. Christopher J. Murphy D. V. M. (2012). Transformation in corneal wound healing and pathology. Vet. Ophthalmol. 12, 25–27. 10.1111/j.1463-5224.2009.00742.x.Meet PMC3290125 19891648

[B21] Kim Y. S. Hong G. Kim D. H. Kim Y. M. Kim Y. K. Oh Y. M. (2018). The role of FGF-2 in smoke-induced emphysema and the therapeutic potential of recombinant FGF-2 in patients with COPD. Exp. Mol. Med. 50 1–10. 10.1038/s12276-018-0178-y PMC6235987 30429461

[B22] King T. E. Pardo A. Selman M. (2011). Idiopathic pulmonary fibrosis. Lancet. 378, 1949–1961. 10.1016/S0140-6736(11)60052-4 21719092

[B23] Klein S. Frohn F. Magdaleno F. Reker-Smit C. Schierwagen R. Schierwagen I. (2019). Rho-kinase inhibitor coupled to peptide-modified albumin carrier reduces portal pressure and increases renal perfusion in cirrhotic rats. Sci. Rep. 9, 1–11. 10.1038/s41598-019-38678-5 30783172 PMC6381202

[B24] Knipe R. Probst C. Ahluwalia N. Shea B. Franklin A. Grasberger P. (2016). ROCK isoforms ROCK 1 and ROCK 2 are critical for the development of pulmonary fibrosis in several different cell specific mechanisms. QJM An Int. J. Med. 109, S2 10.1093/qjmed/hcw118.005

[B25] Knipe R. S. Probst C. K. Lagares D. Franklin A. Spinney J. J. Brazee P. L. (2018). The rho kinase isoforms ROCK1 and ROCK2 each contribute to the development of experimental pulmonary fibrosis. Am. J. Respir. Cell Mol. Biol. 58, 471–481. 10.1165/rcmb.2017-0075OC 29211497 PMC5894496

[B26] Majolée J. Pronk M. C. A. Jim K. K. van Bezu J. S. M. van der Sar A. M. Hordijk P. L. (2019). CSN5 inhibition triggers inflammatory signaling and Rho/ROCK-dependent loss of endothelial integrity. Sci. Rep. 9, 1–12. 10.1038/s41598-019-44595-4 31148579 PMC6544660

[B27] Martin-Medina A. Lehmann M. Burgy O. Hermann S. Baarsma H. A. Wagner D. E. (2018). Increased extracellular vesicles mediate WNT5A signaling in idiopathic pulmonary fibrosis. Am. J. Respir. Crit. Care Med. 198 (12), 1527–1538. 10.1164/rccm.201708-1580OC 30044642

[B28] Meng X. M. Nikolic-Paterson D. J. Lan H. Y. (2016). TGF-β: the master regulator of fibrosis. Nat. Rev. Nephrol. 12 (6), 325–338. 10.1038/nrneph.2016.48 27108839

[B29] Nagai Y. Matoba K. Kawanami D. Takeda Y. Akamine T. Ishizawa S. (2019). ROCK2 regulates TGF-β-induced expression of CTGF and profibrotic genes via NF-κB and cytoskeleton dynamics in mesangial cells. Am. J. Physiol. Ren. Physiol. 317 (4), F839–F851. 10.1152/ajprenal.00596.2018 31364374

[B30] Ng-Blichfeldt J. P. Schrik A. Kortekaas R. K. Noordhoek J. A. Heijink I. H. Hiemstra P. S. (2018). Retinoic acid signaling balances adult distal lung epithelial progenitor cell growth and differentiation. EBioMedicine. 36, 461–474. 10.1016/j.ebiom.2018.09.002 30236449 PMC6197151

[B31] Ng-Blichfeldt J. P. de Jong T. Kortekaas R. K. Wu X. Lindner M. Guryev V. (2019). Tgf-β activation impairs fibroblast ability to support adult lung epithelial progenitor cell organoid formation. Am. J. Physiol. Lung Cell Mol. Physiol. 317 (1), L14–L28. 10.1152/ajplung.00400.2018 30969812

[B32] Noe N. Shim A. Millette K. Luo Y. Azhar M. Shi W. (2019). Mesenchyme-specific deletion of Tgf-β1 in the embryonic lung disrupts branching morphogenesis and induces lung hypoplasia. Lab. Invest. 99, 1363–1375. 10.1038/s41374-019-0256-3 31028279 PMC7422700

[B33] Oenema T. A. Maarsingh H. Smit M. Groothuis G. M. M. Meurs H. Gosens R. (2013). Bronchoconstriction induces TGF-β release and airway remodelling in Guinea pig lung slices. PLoS One. 8 (6), e65580 10.1371/journal.pone.0065580 23840342 PMC3694103

[B34] Ohgiya M. Matsui H. Tamura A. Kato T. Akagawa S. Ohta K. (2017). The evaluation of interstitial abnormalities in group B of the 2011 global initiative for chronic obstructive lung disease (GOLD) classification of chronic obstructive pulmonary disease (COPD). Intern. Med. 56, 2711–2717. 10.2169/internalmedicine.8406-16 28924113 PMC5675931

[B35] Ornitz D. M. Itoh N. (2015). The fibroblast growth factor signaling pathway. Wiley Interdiscip. Rev. Dev. Biol. 4, 215–266. 10.1002/wdev.176 25772309 PMC4393358

[B36] Park J. S. Park H. J. Park Y. S. Lee S. M. Yim J. J. Yoo C. G. (2014). Clinical significance of mTOR, ZEB1, ROCK1 expression in lung tissues of pulmonary fibrosis patients. BMC Pulm. Med. 14 (1), 168 10.1186/1471-2466-14-168 25358403 PMC4233073

[B37] Park J. S. Kim D. H. Shah S. R. Kim H. N. Kshitiz, Kim P. (2019). Switch-like enhancement of epithelial-mesenchymal transition by YAP through feedback regulation of WT1 and Rho-family GTPases. Nat. Commun. 10, 1–15. 10.1038/s41467-019-10729-5 31243273 PMC6594963

[B38] Pireddu R. Forinash K. D. Sun N. N. Martin M. P. Sung S. S. Alexander B. (2012). Pyridylthiazole-based ureas as inhibitors of Rho associated protein kinases (ROCK1 and 2). Medchemcomm. 3, 699–709. 10.1039/c2md00320a 23275831 PMC3531244

[B39] Plikus M. V. Guerrero-Juarez C. F. Ito M. Li Y. R. Dedhia P. H. Zheng Y. (2017). Regeneration of fat cells from myofibroblasts during wound healing. Science 84. 355, 748–752. 10.1126/science.aai8792 PMC5464786 28059714

[B40] Prince L. S. (2018). FGF10 and human lung disease across the Life spectrum. Front. Genet. 9, 1–6. 10.3389/fgene.2018.00517 30429870 PMC6220039

[B41] Puschhof J. Clevers H. (2018). The myofibroblasts’ war on drugs. Dev. Cell. 46, 669–670. 10.1016/j.devcel.2018.09.008 30253163

[B42] Qi X. J. Ning W. Xu F. Dang H. X. Fang F. Li J. (2015). Fasudil, an inhibitor of Rho-associated coiled-coil kinase, attenuates hyperoxia-induced pulmonary fibrosis in neonatal rats. Int. J. Clin. Exp. Pathol. 8, 12140–12150 26722398 PMC4680343

[B43] Rath N. Olson M. F. (2012). Rho-associated kinases in tumorigenesis: Re-considering ROCK inhibition for cancer therapy. EMBO Rep. 13, 900–908. 10.1038/embor.2012.127 22964758 PMC3463970

[B44] Saidi A. Kasabova M. Vanderlynden L. Wartenberg M. Kara-Ali G. H. Marc D. (2019). Curcumin inhibits the TGF-β1-dependent differentiation of lung fibroblasts via PPARγ-driven upregulation of cathepsins B and L. Sci. Rep. 9, 491 10.1038/s41598-018-36858-3 30679571 PMC6345753

[B45] Schiller H. B. Montoro D. T. Simon L. M. Rawlins E. L. Meyer K. B. Strunz M. (2019). The human lung cell atlas: a high-resolution reference map of the human lung in health and disease. Am. J. Respir. Cell Mol. Biol. 61 (1), 31–41. 10.1165/rcmb.2018-0416TR 30995076 PMC6604220

[B46] Shiraishi K. Shichino S. Ueha S. Nakajima T. Hashimoto S. Yamazaki S. (2019). Mesenchymal-epithelial interactome analysis reveals essential factors required for fibroblast-free alveolosphere formation. iScience. 11, 318–333. 10.1016/j.isci.2018.12.022 30639966 PMC6329323

[B47] Tumbarello D. A. Turner C. E. (2006). Hic-5 contributes to transformation through a RhoA/ROCK-dependent pathway. J. Cell. Physiol. 211 (3), 736–747. 10.1002/JCP. 17299801

[B48] Van Dijk E. M. Culha S. Menzen M. H. Bidan C. M. Gosens R. (2017). Elastase-induced parenchymal disruption and airway hyper responsiveness in mouse precision cut lung slices: toward an *ex vivo* COPD model. Front. Physiol. 7, 657 10.3389/fphys.2016.00657 28101062 PMC5209351

[B49] Vigil D. Kim T. Y. Plachco A. Garton A. J. Castaldo L. Pachter J. A. (2012). ROCK1 and ROCK2 are required for non-small cell lung cancer anchorage-independent growth and invasion. Cancer Res. 72, 5338–5347. 10.1158/0008-5472.CAN-11-2373 22942252 PMC3810962

[B50] Villar J. Zhang H. Slutsky A. S. (2019). Lung repair and regeneration in ARDS: role of PECAM1 and wnt signaling. Chest. [Epub ahead of print]. 10.1016/j.chest.2018.10.022 PMC6435939 30392791

[B51] Vuga L. J. Ben-Yehudah A. Kovkarova-Naumovski E. Oriss T. Gibson K. F. Feghali-Bostwick C. (2009). WNT5A is a regulator of fibroblast proliferation and resistance to apoptosis. Am. J. Respir. Cell Mol. Biol. 41 (5), 583–589. 10.1165/rcmb.2008-0201OC 19251946 PMC2778165

[B52] Wang Y. C. Chen Q. Luo J. M. Nie J. Meng Q. H. Shuai W. (2019). Notch1 promotes the pericyte-myofibroblast transition in idiopathic pulmonary fibrosis through the PDGFR/ROCK1 signal pathway. Exp. Mol. Med. 51, 1–11. 10.1038/s12276-019-0228-0 PMC6430797 30902967

[B53] Weiner A. I. Jackson S. R. Zhao G. Quansah K. K. Farshchian J. N. Neupauer K. M. (2019). Mesenchyme-free expansion and transplantation of adult alveolar progenitor cells: steps toward cell-based regenerative therapies. Npj Regen. Med. 4, 1–10. 10.1038/s41536-019-0080-9 31452939 PMC6702233

[B54] Winters N. I. Burman A. Kropski J. A. Blackwell T. S. (2019). Epithelial injury and dysfunction in the pathogenesis of idiopathic PulmonaryFibrosis. Am. J. Med. Sci. 357, 374–378. 10.1016/j.amjms.2019.01.010 31010463 PMC6481315

[B55] Wu X. van Dijk E. M. Bos I. S. T. Kistemaker L. E. M. Gosens R. (2019a). Mesenchymal WNT-5A/5B signaling represses lung alveolar epithelial progenitors. Cells. 8 (10), 1147 10.3390/cells8101147 PMC6829372 31557955

[B56] Wu X. van Dijk E. M. Bos I. S. T. Kistemaker L. E. M. Gosens R. (2019b). Mouse lung tissue slice culture. Methods Mol. Biol., 1940: 297–311. 10.1007/978-1-4939-9086-3_21 30788834

[B57] Xueyang D. Zhanqiang M. Chunhua M. Kun H. (2016). Fasudil, an inhibitor of Rho-associated coiled-coil kinase, improves cognitive impairments induced by smoke exposure. Oncotarget. 7, 78764–78772. 10.18632/oncotarget.12853 27791202 PMC5346675

[B58] Zepp J. A. Morrisey E. E. (2019). Cellular crosstalk in the development and regeneration of the respiratory system. Nat. Rev. Mol. Cell Biol. 20, 551–566. 10.1038/s41580-019-0141-3 31217577 PMC7254499

[B59] Zhang Y. Li X. Qi J. Wang J. Liu X. Zhang H. (2009). Rock2 controls TGFβ signaling and inhibits mesoderm induction in zebrafish embryos. J. Cell Sci. 122, 2197–2207. 10.1242/jcs.040659 19509062

