# Supplementary material for: Rho-Kinase 1/2 Inhibition Prevents Transforming Growth Factor-β-Induced Effects on Pulmonary Remodeling and Repair
Source: Front Pharmacol. 2021 Jan 20;11:609509. doi: 10.3389/fphar.2020.609509 (PMC7855981; doi:10.3389/fphar.2020.609509)
Supplement: Supplementary file 1 [file datasheet1.pdf]

Supplementary Material

Supplement Table 1. Primers used for qRT-PCR analysis.

Gene	Primer sequence	NCBI accession number
Mouse ACTA2	Forward – CTGACAGAGGCACCACTGAA<br>Reverse – CATCTCCAGAGTCCAGCACA	NC_000085.6
Mouse FN	Forward – ACCACCCAGAACTACGATGC<br>Reverse – GGAACGTGTCGTTCACATTG	NC_000067.6
Mouse collagen Iα1	Forward – CACCCTCAAGAGCCTGAGTC<br>Reverse – GTTCGGGCTGATGTACCAGT	NC_000077.6
Mouse 18S	Forward – AAACGGCTACCACATCCAAG<br>Reverse – CCTCCAATGGATCCTCGTTA	NC_000083.5
Human ACTA2	Forward – CCG GGA GAA AAT GAC TCA AA<br>Reverse – GAA GGA ATA GCC ACG CTC AG	NC_000010.11
Human FN	Forward – TCG AGG AGG AAA TTC CAA TG<br>Reverse – ACA CAC GTG CAC CTC ATC AT	NC_000002.12
Human collagen Iα1	Forward – AGC CAG CAG ATC GAG AAC AT<br>Reverse – TCT TGT CCT TGG GGT TCT TG	NC_000017.11
Human WNT2B	Forward – CCG AGA GTG TCA GCA CCA AT<br>Reverse – CTG CCT CTC GGC TAC TTC TG	NC_000001.11
Human WNT-5A	Forward – GGG TGG GAA CCA AGA<br>Reverse – TGG AAC CTA CCC ATC CCA TA	NC_000003.12

Human Axin2	Forward – ACA ACA GCA TTG TCT CCA AGC AGC<br><br>Reverse – GCG CCT GGT CAA ACA TGA TGG AAT	NC_000017.11
Human FGF2	Forward – AAA AAC GGG GGC TTC TTC CT<br><br>Reverse – TGT AGC TTG ATG TGA GGG TCG	NC_000004.12
Human FGF10	Forward – ATG TCC GCT GGA GAA AGC TA<br><br>Reverse – CCC CTT CTT GTT CAT GGC TA	NG_011446.1
Human HGF	Forward – TGC CTG AAA GAT ATC CCG ACA A<br><br>Reverse – GCC TTC TCC TTG ACC TTG GA	NC_000007.14
Human SDHA	Forward – GGG AAG ACT ACA AGG TGC GG<br><br>Reverse – CTC CAG TGC TCC TCA AAC GG	NG_012339.1

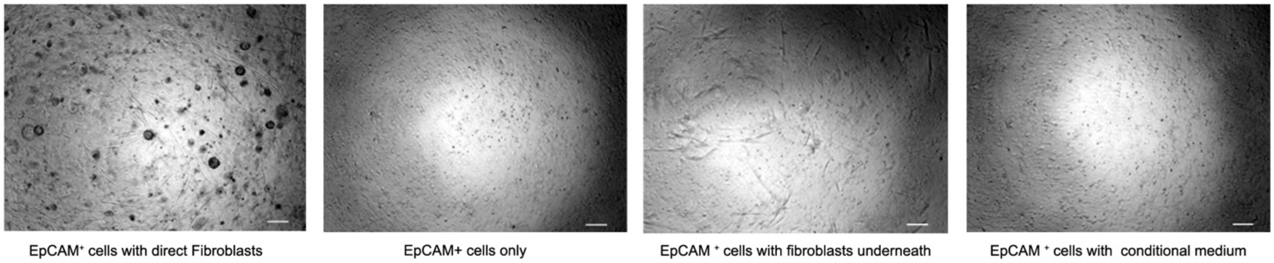

Supplement Figure 1. Four organoid culture systems.

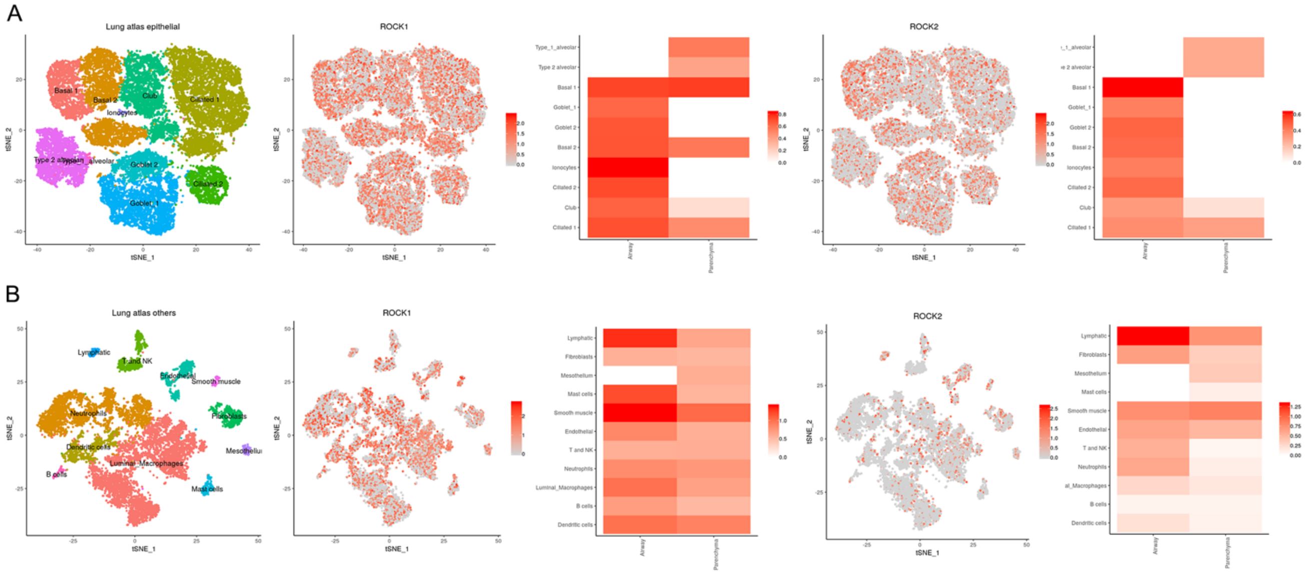

Supplement Figure 2. ROCK1 expresses more than ROCK2 in human lung cells.

(A), ROCK1 and ROCK2 expression in human lung epithelial cells.

(B), ROCK1 and ROCK2 expression in other human lung cells.

All data are from human lung cell atlas (https://asthma.cellgeni.sanger.ac.uk/).

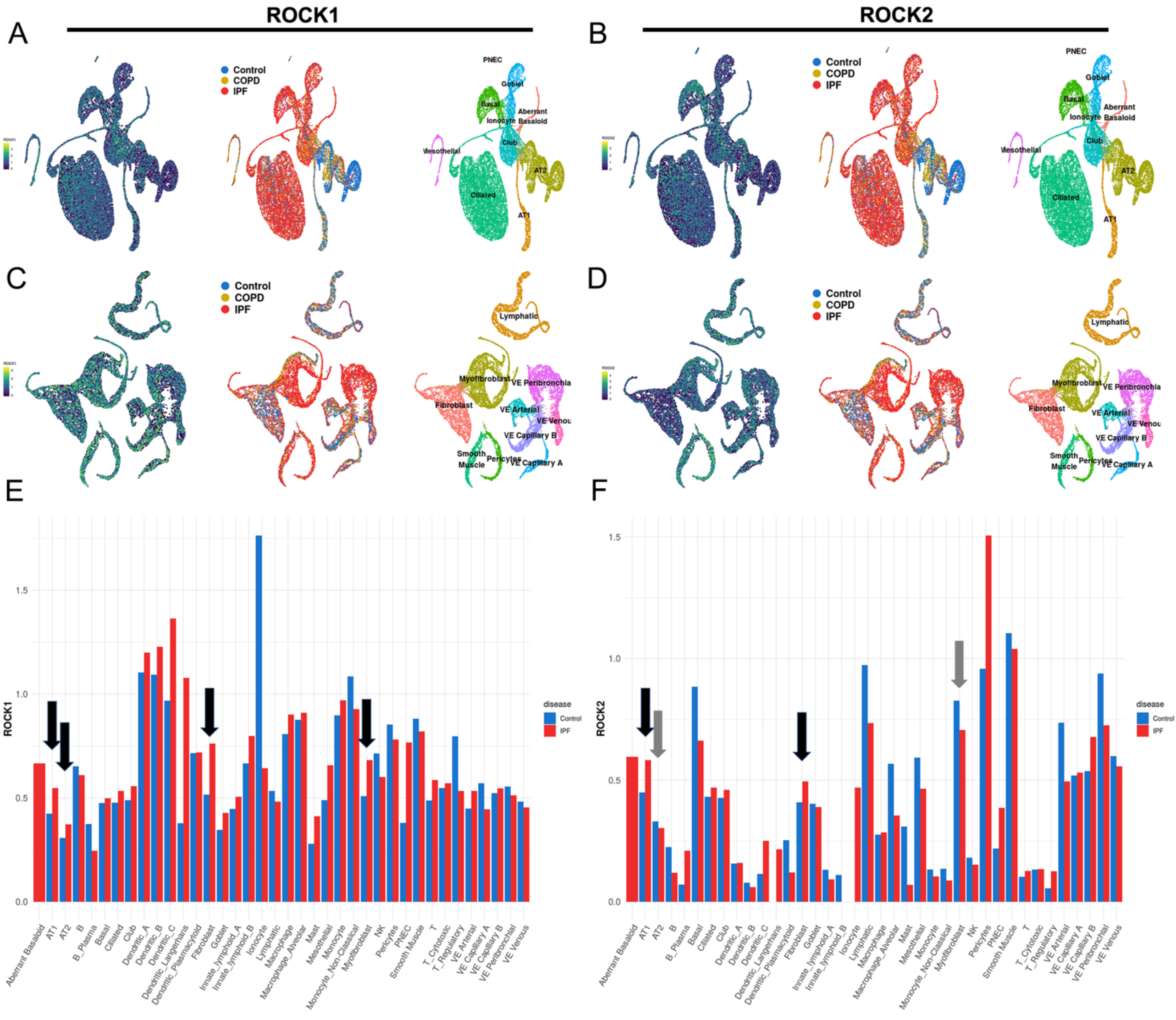

Supplement Figure 3. ROCK1 and ROCK2 expression in the lung.

(A-B), ROCK1 and ROCK2 expression in human epithelial cells. (C-D), ROCK1 and ROCK2 expression in human stromal cells. (E-F), ROCK1 and ROCK2 expression in different cell types in control (blue) and IPF patients (red). All data within this figure are acquired from the database of IPF lung cell atlas (http://www.ipfcellatlas.com/). Control: 29, COPD: 18 patients, IPF: 32 patients
